# Editorial: Basic research on bone development, bone homeostasis, and new strategies on bone regeneration

**DOI:** 10.3389/fphys.2023.1285197

**Published:** 2023-09-15

**Authors:** Ce Shi, Yuji Mishina, Xiaowei Xu, Leiying Miao, Liming Jiang

**Affiliations:** ^1^ Department of Oral Pathology, Hospital of Stomatology, Jilin University, Changchun, China; ^2^ Department of Biologic and Materials Sciences & Prosthodontics, School of Dentistry, University of Michigan, Ann Arbor, MI, United States; ^3^ Hospital of Stomatology, Jilin University, Changchun, China; ^4^ Department of Cariology and Endodontics, Nanjing Stomatological Hospital, Affiliated Hospital of Medical School, Nanjing University, Nanjing, China; ^5^ Department of Paediatric Dentistry, School of Stomatology, China Medical University, Shenyang, China

**Keywords:** bone homeostasis, bone regeneration, stem cell, osteoblast, osteocyte

Bones play crucial roles throughout life. Regardless of their size or shape, they offer structural support for our bodies, shield internal organs and tissues, store vital minerals, and aid in blood cell production. Bone formation occurs through intramembranous or endochondral ossification. Subsequently, bones undergo continuous modeling and remodeling. However, aging, tumors, fractures, and inflammation can lead to bone loss, impacting health. Thus, regenerating lost bone tissue is paramount.

To restore healthy bone tissue that matches the quantity and quality of host bones, a comprehensive understanding of the bone microenvironment in both physiological and pathological contexts is essential. This includes roles of osteoblasts, osteoclasts, osteocytes, and their crosstalk, as well as the contributions from endocrine systems and circulation. Therefore, comprehending the intricacies of bone development and homeostasis is indispensable. The theoretical frameworks from these insights can be harnessed to advance the field of bone regeneration.

This Research Topic covers comprehensive characterization and functions of bone related cells, including bone marrow mesenchymal stem cells, hematopoietic stem cells, osteoblasts, osteoclasts, and osteocytes, among others. These cells participate in bone development, maintenance, remodeling, and regeneration. As editors, we took great delight in assessing captivating articles and reviews. This editorial summarizes primary findings and viewpoints from accepted articles.

Aging is correlated with reduced bone mass and higher osteoporosis risk. Exercise significantly enhance bone metabolism. Studies show that peripheral afferent nerve fibers regulate osteoanabolic activities during mechanical loading. Lee et al. explore the effects of aerobic exercise on bone metabolism and skeletal nerve regeneration. Findings reveal notable improvements in distal femoral and proximal tibial bone parameters, along with a substantial increase in skeletal nerve fiber density, subsequent to aerobic exercise. Additionally, a noteworthy correlation between skeletal nerve densities and trabecular BV/TV has been observed in exercising mice. Aerobic exercise alters microRNA expression profiles, reinforcing its positive effect on bone and skeletal nerve regeneration.

Bone defects resulting from inflammation, trauma, and tumors present a challenge for clinicians. Drawing inspiration from natural bone healing processes, there has been a growing interest in the use of blood-derived products in recent years, with a focus on platelet-rich fibrin (PRF). To address the limitations associated with fresh PRF, lyophilized PRF has been developed. Liu et al. introduced genipin into the lyophilized PRF matrix. The authors demonstrate that genipin-modified lyophilized PRF exhibits superior biomechanics, gradual biodegradation, favorable biocompatibility, and sustained release of growth factors. Genipin-crosslinked lyophilized PRF supports osteoblastic differentiation *in vitro* and bone regeneration *in vivo*.

Osteomyelitis is a progressive inflammatory process leading to bone destruction and necrosis. Earlier studies have identified associations between single nucleotide polymorphisms (SNPs) within *VDR* gene (encoding vitamin D receptor) with susceptibility to inflammatory disorders. Zhao et al. identify significant correlations between rs7975232, rs1544410, and the onset of osteomyelitis. Distinct SNP genotypes are related to varying serological vitamin D levels, potentially influencing VDR protein levels. Elevated levels of vitamin D and VDR have been linked to the inhibition of bacterial growth, facilitation of macrophage functions, and mitigation of ROS-mediated macrophage apoptosis through the VDR-Bmi1 pathway.

The impact of electrical stimulation on bone healing is well acknowledged. However, the precise underlying cellular mechanisms remain unclear. Sahm et al. explore the response of human osteoblasts to varying electric fields over 31 days. The findings indicate that lower electric field upregulates osteogenic markers post-initial stimulation. Notably, both the lower and higher electric fields enhance protein secretion by pre-osteoblasts. Moreover, lower electric field induces the mineralization of the matrix in cultured cells.

Bone fractures profoundly impact quality of life and increase morbidity. Successful bone repair relies on recruiting appropriate skeletal stem cells (SSCs)/progenitors, offering a therapeutic approach for skeletal dysfunction. However, the heterogeneity of SSC populations impedes their applications. Liu et al. reveal transcriptomic heterogeneity in Prx1-expressing SSCs between those derived from muscle and periosteum. Transplantation experiments revealed that periosteal cells, when transplanted onto bone tissue surfaces, differentiated into bone and cartilage cells. However, these cells failed to exhibit similar differentiation potential when transplanted into muscle. Muscle Prx1-SSCs displayed negligible differentiation potential at both transplantation sites. This study underscores the diversity of the Prx1-SSC population, highlighting intrinsic differences among cells from different tissues.

FGF23, produced by osteocytes, regulates phosphate and vitamin D metabolism. Technical challenges in isolating osteocytes together with the notably low levels of Fgf23 gene expression in these cells have hindered the comprehensive investigations of the biological characteristics of osteocytes. Leveraging the knowledge that 1,25-dihydroxyvitamin D_3_ can enhance FGF23 production, Hanai et al. employed single-cell RNA sequencing (scRNA-seq) to profile Fgf23-expressing osteocytes in response to calcitriol (1,25-dihydroxyvitamin D_3_) treatment. The findings reveal that untreated mice exhibit no detectable Fgf23 expression in osteocytes. However, mice treated with calcitriol exhibit Fgf23 expression within a distinct osteocyte subpopulation. These Fgf23-expressing osteocytes also exhibit the expression of Ccnd2, Fn1, Igfbp7, Pdgfa, and Timp1 in response to calcitriol treatment. Additionally, the Fgf23-expressing osteocytes manifest higher levels of Fam20c, Dmp1, and Phex, genes associated with FGF23-related hypophosphatemic diseases. These findings underscore the sensitivity of Fgf23-expressing osteocyte in responding to 1,25-dihydroxyvitamin D_3_ and imply the role of Fgf23-expressing osteocytes in phosphate metabolism.

In summary, investigating bone defect repair processes and understanding key molecules and mechanisms are critical in medical research. The effective recruitment of skeletal stem cells and progenitors for repair pivotal for skeletal disorders. However, the heterogeneity of skeletal stem cell populations has led to a lack of consensus regarding specific cell markers. Despite the challenges, significant progress has been made in identifying potential avenues for bone repair through the exploration of specific gene expressions like Prx1-expressing cells. Additionally, new techniques like single-cell RNA sequencing (scRNA-seq) and transcriptome analysis have unveiled stem cell diversity, site- and function-specific traits. These studies deepen our understanding of bone repair mechanisms and provide novel therapeutic strategies. Investigating the heterogeneity of skeletal stem cells and the functional disparities improves skeletal disorder management, quality of life, and complication reduction. This research provides valuable insights that advance the medical field.

